# Flow diversion of cerebral aneurysms in Type I osteogenesis imperfecta: A case report of the first two treatments in humans

**DOI:** 10.1177/2050313X241274243

**Published:** 2024-08-24

**Authors:** David A Zarrin, Jessica K Campos, Benjamen M Meyer, Alexander S Himstead, Fahad Laghari, Jonathan C Collard de Beaufort, Kiarash Golshani, Narlin B Beaty, Matthew T Bender, Geoffrey P Colby, Alexander L Coon

**Affiliations:** 1 David Geffen School of Medicine, University of California Los Angeles, Los Angeles, CA, USA; 2 Department of Neurological Surgery, University of California Irvine, Orange, CA, USA; 3 College of Medicine, University of Arizona, Tucson, AZ, USA; 4 Carondelet Neurological Institute, St. Joseph’s Hospital, Tucson, AZ, USA; 5 State University of New York Upstate Medical University, Syracuse, NY, USA; 6 Florida State University, Tallahassee Memorial Hospital, Tallahassee, FL, USA; 7 Department of Neurosurgery, University of Rochester, Rochester, NY, USA; 8 Department of Neurosurgery, University of California Los Angeles, Los Angeles, CA, USA

**Keywords:** Cerebral aneurysm, flow diversion, osteogenesis imperfecta

## Abstract

Osteogenesis imperfecta (OI) predisposes individuals to easy bone fracture, vessel fragility, and platelet dysfunction. We report the first known case of neurointerventional treatment with flow diversion of intracranial aneurysms in a patient with OI. A 62 year-old female with known OI Type I, history of >40 lifetime bone fractures and hypertension, underwent workup for transient ischemic attacks revealing a 4-mm right A1 segment aneurysm in 2016. Perioperative dual antiplatelet therapy was aspirin 81 mg and clopidogrel 37.5 mg daily. Tri-axial access was utilized to deploy a 3.5 × 16-mm Pipeline Flex device without complication. Two-month follow-up revealed Raymond I (O’Kelly Marotta I) obliteration of the aneurysm. Five-year follow-up revealed a de novo left-sided 3-mm A1–A2 junction aneurysm. A 4 × 12-mm Surpass Evolve was placed without complication. Six-month follow-up revealed Raymond I (O’Kelly Marotta I) obliteration of the second aneurysm. The patient remained asymptomatic at all follow-up visits.

## Introduction

Osteogenesis imperfecta (OI) is an autosomal dominant connective tissue disorder occurring in approximately one per 20,000 live births.^1^ This condition is caused primarily by mutations in *COL1A1* and *COL1A2* genes, which result in defective synthesis of type I collagen, a structural protein contributing to the mechanical integrity of bone, tendons, skin, and blood vessel walls, among other tissues. Afflicted individuals are therefore predisposed to a myriad of diseases including easy bone fracture, vessel fragility, and platelet dysfunction.

Numerous reports have described vascular pathology in association with OI. Vessel dissections in OI patients have been repeatedly reported, particularly dissections of the carotid artery, cervical arteries, and aorta,^2,3^ suggesting underlying vessel fragility may be a component of the disorder. Furthermore, there is a significant association between *COL1A* mutations and the formation of intracranial aneurysms,^4^ and it has been proposed that underlying collagen defects in OI may contribute to the formation of cerebral aneurysms.^5,6^ Despite this genetic association, the role of OI as a causative agent in cerebral aneurysm formation remains unproven, as the overall incidence of intracranial aneurysms in patients with OI is still unknown.

Several reports in the literature describe the treatment of cerebral aneurysms in OI patients. Among ruptured and unruptured aneurysms in OI, previously employed treatment modalities include only open microsurgical clipping^6^ and endovascular coiling.^7^ To our knowledge, there are no studies which describe the use of flow diversion for the treatment of a cerebral aneurysm in an OI patient. In the present case illustration, we describe the first known cases of flow diversion embolization of intracranial aneurysms in a patient with OI. Ethical approval was obtained from the MetroWest Medical Center Institutional Review Board (IRB# 2020-039).

## Case

### Medical history

Written informed consent was obtained from the patient for their anonymized information to be published in this article. The patient is female in her 60s with a history of OI Type I and hypertension. She was first diagnosed with OI Type I after undergoing genetic testing at a young age following a series of bone fractures. Over her lifetime, the patient sustained >40 bone fractures and exhibited a tendency to bruise and bleed easily. This patient is a lifetime nonsmoker and has no history of subarachnoid hemorrhage. Of note, her medications included estradiol. She has no neurological deficits at baseline.

#### First case presentation

**Figure 1.**
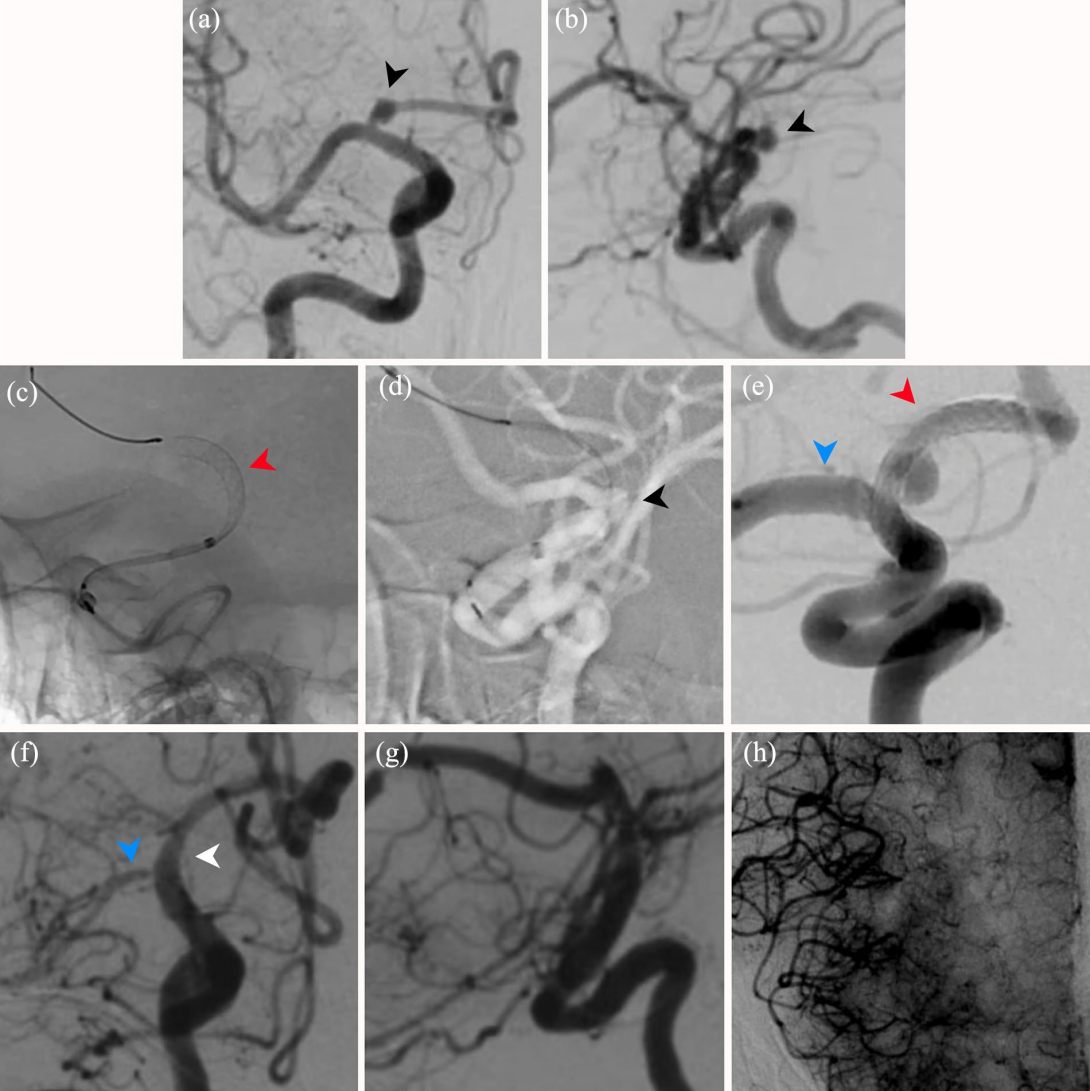
Stage 1: Preoperative diagnostic cerebral angiography demonstrating anterior-posterior (AP) (a) and lateral (b) projections of the 4-mm right A1 segment aneurysm (black arrowheads). Middeployment lateral unsubtracted (c) view of PED Flex deployment (black arrowhead) and intermediate access catheter, Catalyst5. Roadmap (d) view following PED Flex deployment across the neck of the aneurysm (black arrowhead). Postoperative AP (e) projection of the PED (red arrowhead) deployed across the neck of the aneurysm, jailing the right MCA (blue arrowhead demonstrating patency of right MCA). Five-year follow-up angiogram AP (f) and lateral (g) view demonstrating complete aneurysm obliteration (white arrowhead) and reduced MCA (blue arrowhead) filling with no in-stent thrombosis, displacement, pseudoaneurysm, or dissection in the parent vessel, and (h) robust MCA pial collateralization from the ACA. PED Flex: Pipeline Embolization Device Flex.

In 2016, the patient suffered from a transient ischemic attack initially presenting with aphasia. On subsequent digital subtraction angiography workup, a saccular aneurysm measuring 4-mm in maximum diameter was incidentally discovered on the right A1 segment, associated with right cavernous internal carotid artery (ICA) grade III^8^ tortuosity (Figure 1(a) and (b)). The patient was evaluated by our endovascular and cerebrovascular team, and the decision was made to treat the aneurysm with Pipeline Embolization Device Flex (PED Flex; Medtronic Neurovascular, Irvine, CA, USA) flow diversion given the aneurysm position, size, morphology, case complexity, and the patient’s comorbidities.

### Second case presentation

**Figure 2.**
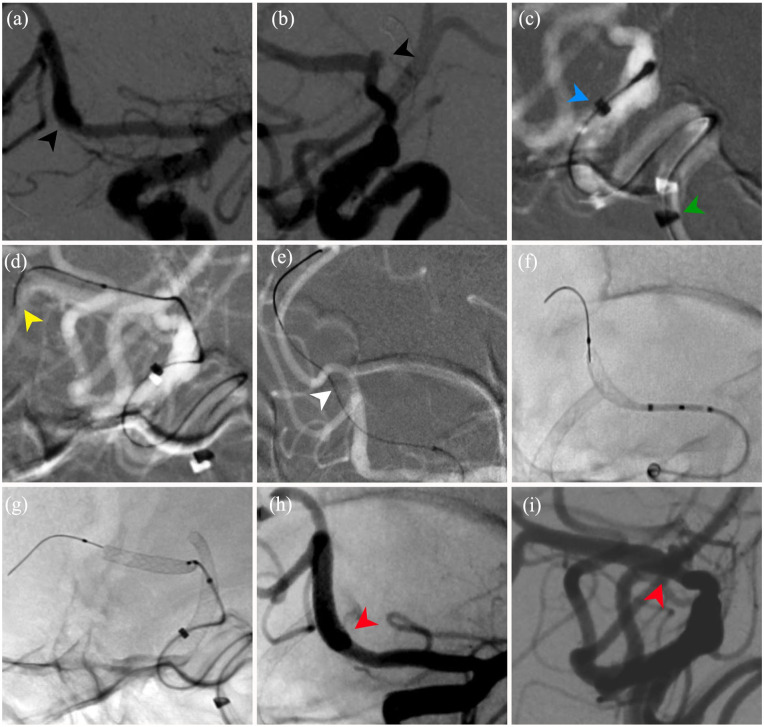
Stage 2: Five-year follow-up angiogram AP (a) and lateral (b) views demonstrating de novo left A1–A2 junction aneurysm (black arrowheads). Intraoperative lateral roadmap views showing (c) the guide catheter in the cavernous ICA (green arrowhead) and distal access catheter in the supraclinoid ICA (blue arrowhead); (d) the SL-10 microcatheter containing the 200 cm floppy tip guide wire (yellow arrowhead) distal to the aneurysm after the delivery assist catheter was removed; (e) the XT-27 microcatheter inserted over the 300 cm floppy tip guide wire with straightening of the vessel architecture (white arrowhead). Intraoperative unsubtracted view demonstrating the Surpass Evolve in position during (f) and at near-maximal (g) deployment. Postdeployment subtracted AP (h) and lateral (i) views showing Surpass Evolve across the aneurysm neck demonstrating complete deployment with no hemorrhage, in-stent thrombosis, pseudoaneurysm, or dissection, and demonstrating complete patency of distal vasculature. ICA: internal carotid artery.

In 2021, a separate de novo left-sided A1–A2 junction saccular aneurysm measuring 3-mm in maximum diameter was discovered on serial vascular imaging, as well as left cervical ICA tortuosity (Figure 2(a) and (b)). The patient was again evaluated by our team and an endovascular approach, utilizing the Surpass Evolve flow diverter device (Stryker, Neurovascular, Fremont, CA, USA) was carefully planned, with both patient and physician acknowledgment again that use of flow diversion in the distal circulation is an off-label use of the device.

### Treatment

Both interventions were performed under biplanar flat panel x-ray fluoroscopy with the patient under general anesthesia. A standard heparin flush was maintained throughout both interventions and the procedures were performed under heparin bolus conditions (4000 Units IV bolus upon access). Glycoprotein II2bIIIA antagonists at a half-cardiac intraarterial bolus followed by postprocedure maintenance drip at full cardiac dosing were utilized postdeployment to prophylax against platelet aggregation in the small vessels of the patient’s distal circulation.^9^

#### Intervention #1

This was an elective PED Flex embolization of the patient’s right A1 segment aneurysm. Dual-antiplatelet therapy (DAPT) consisting of aspirin 81 mg and clopidogrel 37.5 mg daily was initiated 7 days prior to the procedure as previously described by our group.^10^ Preoperative platelet reaction unit (PRU) was 259. A tri-axial catheter access system was used for femoral access and consisted of a guide catheter (Infinity; Stryker Neurovascular), a distal access catheter (Catalyst 5; Stryker Neurovascular), and a microcatheter (VIA27; Sequent Medical/MicroVention Terumo, Tustin, CA, USA) over a guidewire. The guide catheter and then the distal access catheter were advanced to their final positions of the distal petrous and supraclinoid segments of the right ICA, respectively. The VIA27 and guidewire were subsequently advanced into the right distal anterior cerebral artery ( ACA) and a 3.5 × 16 mm PED Flex was successfully deployed across the neck of the aneurysm, jailing the M1 segment in the process (Figure 1(c)–(e)). The PED Flex delivery wire was recaptured without issue. The procedure was completed without complication other than a mild proximal A1 segment spasm which was treated with intraarterial verapamil administration. The patient’s systolic blood pressure was maintained at 160 mmHg for 24-h postembolization and then gradually reduced to minimize the risk for ischemic complication associated with the jailed M1 segment.^11^ Immediate postdeployment angiography demonstrated complete neck coverage and contrast stasis of the aneurysm well into the capillary phase. There were no perioperative complications. A standard six-month regimen of DAPT was initiated, and the patient was discharged home after one night of monitoring in the neurocritical care unit (NCCU).

#### Intervention #2

This was an elective Surpass Evolve embolization of the patient’s left A1–A2 junction aneurysm. DAPT consisting of ticagrelor 60 mg BID and aspirin 81 mg QD was initiated 7 days prior to the procedure. The patient’s PRU was 59 and was within the therapeutic window. A tri-axial catheter access system was used for femoral access and consisted of a guide catheter (Tracstar; Imperative Care, Campbell, CA, USA), a distal access catheter (Catalyst 5) with a delivery assist catheter (Offset; Stryker Neurovascular), and a microcatheter (XT27; Stryker Neurovascular, and Excelsior SL-10; Stryker Neurovascular) over a guidewire. The guide catheter was first placed to its working position in the vertical cavernous ICA (Figure 2(c)). The distal access catheter was subsequently advanced to the supraclinoid ICA using a delivery assist catheter (Offset) to minimize vessel trauma. The delivery assist catheter was then removed and an Excelsior SL-10 microcatheter and Transcend (Stryker Neurovascular) 200 cm floppy tip guidewire were then used to navigate the highly tortuous distal ICA and cross the aneurysm (Figure 2(d)). The Transcend 200 cm guidewire was then exchanged within the SL-10 for a stiffer Transcend 300 cm floppy tip guidewire. The SL-10 was then exchanged out, and the XT-27 microcatheter was tracked across the aneurysm into the A3 segment using the stiff 300 cm Transcend guidewire for support to minimize vessel traction (Figure 2(e)). After confirming patency with the XT-27 in its most distal position, a 4 × 12 mm Surpass Evolve flow diverter was pushed through the XT-27 and deployed across the neck of the aneurysm (Figure 2(f) and (g)). The device was successfully deployed within the 1.5-mm diameter parent vessel and subsequent control angiography demonstrated complete neck coverage and contrast stasis within the aneurysm (Figure 2(h) and (i)). There were no perioperative complications.A 6-month regimen of DAPT (Ticagrelor) was initiated, and the patient was discharged home after two nights of monitoring in the critical care unit.

#### Outcome and follow-up of intervention #1

Angiographic follow-up two-months postoperatively revealed Raymond I obliteration of the aneurysm with robust ACA pial collateralization to the middle cerebral artery (MCA) territory (Figure 1(f)–(h)). The patient was asymptomatic with no neurological deficit on clinical evaluation. Five-year follow-up angiography revealed the de novo left-sided 3-mm A1–A2 junction saccular aneurysm and stable right MCA collateralization with continued obliteration of the A1 segment aneurysm (Figure 2(a) and (b)).

#### Outcome and follow-up of intervention #2

**Figure 3.**
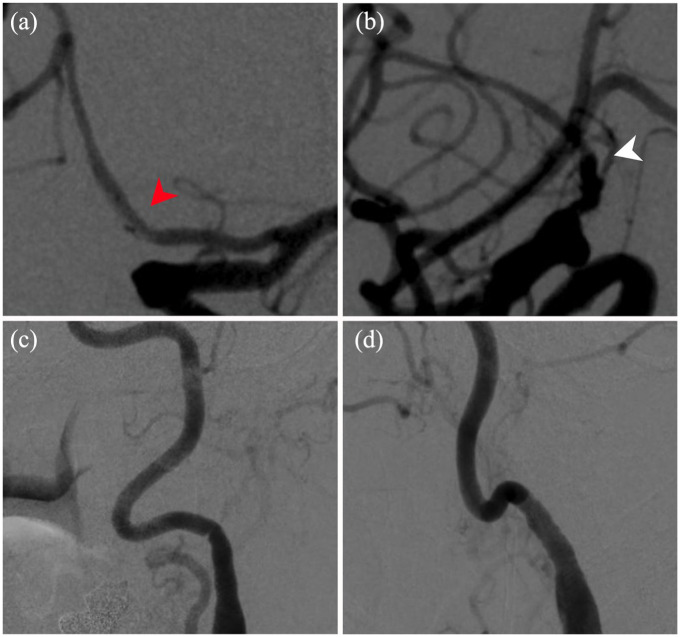
Six-month follow-up subtracted AP (a) and lateral (b) views demonstrating the Surpass Evolve in place (red arrowhead), complete resolution of aneurysm (white arrowhead), and no in-stent thrombosis, pseudoaneurysm, or dissection. Subtracted (c) AP and (d) lateral views of the left internal carotid artery demonstrating no evidence of dissection.

Six-month angiographic follow-up revealed Raymond I aneurysm obliteration (Figure 3(a) and (b)). The left cervical ICA demonstrated no evidence of vessel trauma or dissection. Clinically, the patient remained neurologically intact (Figure 3(c) and (d)).

## Discussion

Literature guiding the endovascular management of cerebral aneurysms in OI is limited to a few case reports of coiling and is absent altogether as it pertains to flow diversion. Thus, the decision to treat both aneurysms with an endovascular approach as opposed to open surgical repair was made only after a careful consideration of the patient’s comorbidities and the perceived procedural complexities. The risks of vessel damage, easy bone fracture, poor wound healing, and medically prolonged blood loss from an open surgical repair were weighed against the risk of vessel damage from endovascular manipulation. The endovascular approach was ultimately selected given the patient’s extensive history of bone fragility, and the fear of unexpected bleeding during open surgery necessitating transfusion as has been reported in OI.^12^ Further, recent advances in catheter access systems allow for safer access to vessels within the Circle of Willis. Only two reports of Type I OI have been identified, one of which had a dissecting superior cerebellar artery successfully repaired via coil, and another that had an aneurysm of the basilar and vertebrobasilar artery, which were successfully repaired via coil and stent coil, respectively.^13,14^

Known predisposition to carotid artery dissection and systemic vessel fragility^2,3^ in OI necessitated a carefully planned endovascular access technique in this patient. A delivery assist catheter, Offset, was used to minimize distal access catheter tip-trauma when navigating tortuous cavernous and clinoid ICA segments, and a Transcend guidewire and microcatheter exchange was performed to mechanically support the advancement of the bulkier XT-27 delivery catheter across a tortuous distal ICA. As previously described, success using a tri-axial system in a patient with extremely fragile vessels due to comorbid Loeys–Dietz syndrome was performed and replicated here.^15^ This experience informed our selection of an analogous tri-axial system in the present interventions. The described use of Offset and a Transcend exchange in combination with a tri-axial system allowed for a gradual evolution of stiffness of the catheter system and therefore minimized mechanical stress on conduit vessels.

The Surpass Evolve (Stryker Neurovascular) embolization device was selected for the second intervention for multiple reasons. First, Surpass Evolve is a single-layer cobalt chromium flow diverter, making it less thrombogenic in our experience than other nitinol-based flow diverters available in the United States such as Flow-Redirection Intraluminal Device (Microvention, Aliso Viejo, CA, USA), which are more susceptible to in-stent stenosis. We intentionally oversized the implanted device as is commonly done in perforator rich regions such as the anterior communicating artery, in order to reduce percentage metal coverage (flow diversion dose) with perceived better rates of perforator preservation. Finally, the Surpass Evolve design simplifies resheathing compared to its counterparts: Surpass Evolve can be directly pulled back due to its softer winglets. In contrast, PED requires wire recapture and is generally associated with heightened mortality in older patients when used off-label.^16^ Although the Surpass Evolve requires the use of an 0.027″ catheter system, this is something that has long been performed safely in proximal anterior cerebral artery aneurysm flow diversion.^17^

Close attention to appropriate anti-platelet therapy response was of critical importance in this patient given the predisposition to platelet dysfunction in OI.^18,19^ Although debate still surrounds the effect of OI on platelet function, there have been numerous reports suggesting platelet function is inhibited and OI patients frequently report easy bleeding and bruising.^19,20^ Ticagrelor was chosen over clopidogrel for the second intervention in line with contemporary thought for its more predictable PRU response profile. This ensured an optimal preoperative PRU and avoided clopidogrel half-dosing altogether. Our group previously showed that safe flow diversion in sub-2 mm vessels is achievable without significant concern for acute stent thrombosis or in-stent stenosis.^21^ Here, we administered intraoperative glycoprotein II2bIIIA antagonists to further minimize the risk of acute stent thrombosis given uncertainty surrounding the state of this patient’s primary hemostatic response. After each procedure, intravenous heparin was allowed to drift off without reversal and the patient was maintained on DAPT for 6 months and subsequently weaned to a single agent over the ensuing 6 months.

Flow diversion in this patient offered several distinct advantages over coil embolization. In the context of vessel fragility, accessing the aneurysm sac with coils introduces the additional risk of perforating a thin-walled aneurysm. Small aneurysms of the ACA like those under consideration are in fact known to be high-risk for rupture.^22^ Furthermore, flow diversion facilitates simultaneous aneurysmal exclusion and reconstruction of the parent vessel, whereas it can be argued that coiling only accomplishes the former. In an OI patient with inherent vessel fragility, addressing the root of the problem with flow diversion may tend to offer a curative solution as seen here, whereas coiling would more likely be prone to recur. With technologically advanced cerebrovascular access catheters, many of the challenges unique to deploying flow diverters in tortuous and distal vessels can now be overcome, and the benefits of such a therapy can be reaped more often, even in higher-risk patients such as those with OI.

There are several key insights to be drawn after endovascularly managing two cerebral aneurysms in a patient with OI, a disease known to predispose to vessel fragility. We demonstrate that with careful use of endovascular catheter technique and a judicious antiplatelet regimen, one can safely perform endovascular flow diversion in OI patients. Furthermore, endovascular flow diversion should be considered when faced with the rare cerebral aneurysm in a patient suffering from a connective tissue disorder such as OI. Finally, we highlight that flow diversion reduces the risk of iatrogenic aneurysmal perforation and better facilitates parent vessel reconstruction relative to coiling, making it ideal for treating aneurysms with potentially elevated rupture risk. Use of a flow diverter does, however, necessitate DAPT given its endoluminal mechanism and carries risk of perforator infarction which must be mitigated by proper sizing and selection of the flow diversion device.

## Conclusion

This report describes the first two distinct endovascular aneurysm flow diversion treatments in a patient with Type I OI. These cases demonstrate that by using careful endovascular catheter technique and a judicious antiplatelet regimen, one can safely perform endovascular flow diversion in OI patients. In summary, this suggests that endovascular flow diversion should be considered when faced with the rare cerebral aneurysm in a patient suffering from a connective tissue disorder such as OI.
